# Probiotic Properties of *Lactococcus lactis* Strains Isolated from Natural Whey Starter Cultures

**DOI:** 10.3390/foods13060957

**Published:** 2024-03-21

**Authors:** Ida De Chiara, Rosangela Marasco, Milena Della Gala, Alessandra Fusco, Giovanna Donnarumma, Lidia Muscariello

**Affiliations:** 1 Department of Environmental, Biological and Pharmaceutical Sciences and Technologies (DiSTABiF), Università degli Studi della Campania Luigi Vanvitelli, Via Vivaldi 43, 81100 Caserta, Italy; ida.dechiara@unicampania.it (I.D.C.); rosangela.marasco@unicampania.it (R.M.); milena.dellagala@unicampania.it (M.D.G.); 2 Department of Experimental Medicine, Section of Microbiology and Clinical Microbiology, Università degli Studi della Campania Luigi Vanvitelli, Via de Crecchio No. 7, 80138 Naples, Italy; alessandra.fusco@unicampania.it (A.F.); giovanna.donnarumma@unicampania.it (G.D.)

**Keywords:** *Lactococcus lactis*, lactic acid bacteria, probiotic bacteria, *Salmonella* Typhimurium, EIEC

## Abstract

*Lactococcus lactis* is a lactic acid bacterium (LAB), generally recognized as safe, and has been widely used in the food industry, especially in fermented dairy products. Numerous studies have evaluated the technological and probiotic properties of lactococci; however, few studies have reported the probiotic characteristics of *L. lactis* strains isolated from dairy products. In this work, probiotic potential, including survival in simulated gastric juice, tolerance to bile salts, hydrophobicity, and auto- and co-aggregation, was evaluated in *L. lactis* strains from natural whey starter cultures. The results highlighted the potential probiotic properties of some strains under study, which showed high values of hydrophobicity and auto-aggregation and low values of co-aggregation with the tested pathogenic strains. In addition, studies of safety parameters, such as antibiotic susceptibility and haemolytic activity, confirmed the safety status of all strains under study. Finally, the four most promising strains were investigated for their ability to inhibit the enteroinvasive *Escherichia coli* (EIEC) and *Salmonella* Typhimurium adhesion to epithelial cells, using a model of co-cultured epithelial cells. The results demonstrated that *L. lactis* strains A3-A5-I4-I7 showed the ability to compete with pathogens as well as the ability to exert a protective effect on cells previously infected with *E. coli* or *S.* Typhimurium. The identification of new probiotic LAB strains from dairy products aims to produce novel functional foods.

## 1. Introduction

Lactic acid bacteria (LAB) are one of the most important health-promoting groups in the human intestinal microbiota. Their protective role within the gut consists of producing in situ antimicrobial compounds, balancing the composition of intestinal commensal microorganisms, and out-competing invading pathogens for ecological niches and metabolic substrates. Traditionally present in dairy products, LAB account for a variety of different characteristics and qualities of the final products, assisting with health maintenance. Indeed, several LAB adopted in the dairy industry as starters are classified as probiotics due to their ability to prevent and treat diseases and modulate immune responses in the host [1,2]. Fermented milk, cheese, yogurt, and kefir are primary sources of probiotic LAB and their consumption has been shown to be associated with beneficial health effects [3,4,5]. An interesting study demonstrated that, during milk fermentation, some lactobacilli are able to release peptides with a high affinity for ACE, the host entry receptor of the SARS-CoV-2 virus [6]. Considering that SARS-CoV-2 infection is associated with immune dysfunction and gut microbiota alterations, the administration of probiotics and/or prebiotics could represent a new and promising therapy for the prevention and treatment of SARS-CoV-2 infection [7,8]. In recent years, several works have suggested the therapeutic role of probiotic LAB in neurodegenerative diseases through gut–brain axis communication [9,10,11]. Indeed, the probiotic intestinal flora produces a large number of neuroactive molecules and metabolites such as short-chain fatty acids (SCFAs), which can have neuroprotective effects, thereby improving the brain’s cognitive functions [12,13]. In addition, the use of health-promoting LAB for the production of postbiotics is also garnering great interest [14,15]. They are water-soluble products derived from microbial metabolism or by-products of bacterial cells after lysis. Considering the probiotic potential of LAB isolated from food products [16], Agagunduz and co-workers [17] proposed a novel concept for the food–gut-health axis. Currently, there is a continuous increase in requests from consumers for natural foods with beneficial characteristics. For this reason, the search for new and beneficial strains with probiotic, biochemical, and technological properties is of primary importance for the food industry. However, to be defined as probiotics, microorganisms must possess certain characteristics. Some criteria for selecting bacteria to be used as probiotics include survival of the probiotic microorganisms through the gastrointestinal tract, ability to adhere to the intestinal epithelium, the capacity for auto-aggregation and co-aggregation, and high cell surface hydrophobicity. In addition, the ability of probiotics to produce molecules with antimicrobial activity and surface molecules capable of adhering to the host’s extracellular matrix gives them the ability to counteract and prevent the adhesion and colonization of pathogenic bacteria thanks to competitive exclusion mechanisms [18,19]. All these features provide advantages for bacteria to colonize the intestine [20].

The genera *Lactobacillus* and *Bifidobacterium* include the largest number of probiotic strains, although many studies have reported the probiotic properties of several *Lactococcus lactis* strains [21,22,23,24]. Due to its historical use in food fermentation, *Lactococcus lactis* is “generally recognized as safe” (GRAS) and has been proposed as a possible vehicle to deliver therapeutic molecules in the gastrointestinal tract [25]. Anti-inflammatory properties have been described for some natural *L. lactis* isolates, whereas the ability to modulate the intestinal microbiota has been reported for an *L. lactis* strain obtained from a fermented milk product [26,27]. Yerlikaya (2019) studied the biochemical, technological, and probiotic properties of *L. lactis* strains isolated from raw milk and kefir grains [28]. The dairy industry uses standardized procedures involving the addition of starter lactic acid bacteria (SLAB) which, together with the microbiota of the raw milk used, determine the properties of the derived products [29,30,31]. However, several typical regional cheeses are often the product of artisanal activities that use natural whey starters (NWS) with high and undefined microbial diversity. Artisanal procedures ensure specific nutritional, organoleptic, and physical properties of the derived cheese [32,33,34,35]. Recently, the microbial community of NWS cultures of cow milk for the production of caciocavallo and buffalo milk for the production of mozzarella, both from artisanal farms, has been described [36]. This study aims to evaluate the probiotic potential of *L. lactis* strains, which were previously isolated from the above-mentioned food matrices. Due to the strain-specific effects of probiotic characteristics, there is great interest in isolating new probiotic candidates from natural sources characterized by high microbial diversity, such as NWS cultures. The selection of new LAB probiotic strains from dairy products is important for the production of novel functional foods with health-related properties.

## 2. Materials and Methods

### 2.1. Bacterial Strains, Media, and Growth Conditions

The *Lactococcus lactis* subsp. *lactis* strains (A3, A5, B1, D1, D3, I1, I4, and I7) used in this study were previously isolated from natural whey starter cultures and their different RAPD profiles were determined [36]. *Lactococcus* strains were grown at 30 °C in ESTY broth supplemented with 1% (*w*/*v*) lactose. For the detection of co-aggregation and antimicrobial activity, the *Escherichia coli* DH5α (ATCC13762), *Salmonella* Typhimurium (ATCC14028), *Shigella sonnei* (ATCC25931), *Staphylococcus aureus* (ATCC6538), *Listeria monocytogens* (ATCC7644), *Pseudomonas aeruginosa* (DSM 1117), and *Enterococcus hirae* (DSM 1018) pathogenic bacteria were used. The first four strains were grown in LB medium, whereas *P. aeruginosa* and *E. hirae* were grown in tryptic soy broth (TSB). *L. monocytogenes* was instead cultured in BHI medium. Pathogenic bacteria were grown at 37 °C. All media were supplied by Condalab (Madrid, Spain). *Salmonella enterica* subsp. *enterica* serovar Typhimurium (ATCC^®^ 14028GFP™) and enteroinvasive *Escherichia coli* (EIEC-ATCC^®^ 43893™), used in the adhesion experiments, were cultured in tryptic soy broth (TSB-OXOID) and in brain heart infusion (BHI-OXOID), respectively, at 37 °C overnight.

### 2.2. Resistance to Simulated Gastrointestinal Conditions

To determine the tolerance of each strain to simulated gastric juice, the method of Talib and co-workers [37] was used with some modifications. Briefly, 4 mL of overnight culture was centrifuged at 10.000 rpm for 5 min and the cells were re-suspended in phosphate-buffered saline solution (PBS) at pH 6.5 to obtain an optical density of 0.6 at 600 nm (OD_600nm_). Afterwards, saline cell suspensions were treated with phosphate-buffered saline solution containing 0.3% (*w*/*v*) pepsin (Merck, Darmstadt, Germany), adjusted to pH 2.0 and pH 3.0 with HCl, and incubated for 3 h at the specific growth temperatures of the analysed strain. The pH tolerance of the cells was determined by enumerating the viable cells on agar plates and expressed as a percentage of viable cells after treatment versus total cells.

For tolerance to pancreatic juice, cell suspensions, obtained as previously described, were treated with phosphate-buffered saline solution containing 0.3% (*w*/*v*) oxgall (Merck, Darmstadt, Germany) and incubated for 3 h. Bile tolerance was estimated by enumerating viable cells on agar plates and comparing the viable cell counts in the presence and absence of bile (oxgall). The percentage of survival was expressed as described above.

### 2.3. Auto-Aggregation and Co-Aggregation Assays

Specific cell–cell interactions were determined using auto-aggregation and co-aggregation assays [38,39]. Bacteria were grown for 18 h, and the cells were then harvested by centrifugation at 3750× *g* for 20 min at room temperature, washed twice with PBS, and resuspended in the same buffer to obtain 0.6 OD_600nm_. For the auto-aggregation assay, each bacterial suspension was vortexed for 10 s and incubated for 24 h at 30 °C without agitation. The absorbance of the supernatant was measured at 600 nm after 2, 4, 6, and 24 h of incubation using a UV-6300PC Double Beam Spectrophotometer (VWR, Milan, Italy). The auto-aggregation percentage was calculated as follows:

Auto-aggregation (%) = (1 − A_t_/A_0_) × 100 (1)

where A_t_ is the absorbance after 2-4-6 or 24 h;

A_0_ is the absorbance at time 0.

For the co-aggregation assay, equal volumes (2 mL) of the probiotic and pathogenic strain cell suspensions (OD_600_ = 0.6) were mixed, vortexed for 10 s, and incubated at 30 °C without agitation. The absorbance of the supernatant was measured at 600 nm after 24 h of incubation. The co-aggregation percentage was calculated as follows:

Co-aggregation (%) = [1 − A_mix_/(A_probiotic_ + A_pathogen_)/2] × 100 (2)

where A_mix_ is the absorbance of the mix (probiotic + pathogen);

A_probiotic_ is the absorbance of the probiotic;

A_pathogen_ is the absorbance of the pathogen.

### 2.4. Hydrophobicity Assay

Bacterial adhesion to hydrocarbons was assessed using the method described by Oliveira et al. [40]. Bacterial cells, grown in the appropriate medium for 18 h, were centrifuged at 6000× *g* for 5 min at room temperature, washed twice with PBS, and adjusted to an OD_600nm_ of 0.6 with 0.1 M KNO_3_ (pH 6.2) (A_0_). After the addition of 16% xylene, cell suspensions were maintained for 10 min at room temperature. The mixture was vigorously stirred and incubated for 30 min for phase separation. The aqueous phases were collected for optical density measurements (A_t_). The percentage reduction in the optical density was then calculated as follows:

Hydrophobicity (%) = (1 − A_t_/A_0_) × 100 (3)

where A_t_ is the absorbance after the mix;

A_0_ is the absorbance at time 0.

### 2.5. Cell Cultures

Colorectal adenocarcinoma Caco-2 cells (ATCC HTB-37^™^) and mucus-secreting colon epithelial HT29-MTX-E12 cells (Sigma Aldrich, Darmstadt, Germany) were cultured separately, using Dulbecco’s modified Eagle’s medium (DMEM, Gibco, Milan, Italy) supplemented with 10% foetal bovine serum, 100 IU/mL penicillin, 100 mg/mL streptomycin, and 2 mM glutamine, at 37 °C in a modified atmosphere of 5% CO_2_. The co-cultures were set up in a 12-well Transwell^®^ (Corning, NY, USA) with a 12 mm polycarbonate insert and 0.4 µm pores, plating the cells in the basolateral compartment in a 7:3 Caco:HT29-MTX ratio, and carried out for 21 days to obtain full differentiation, changing the medium every 2 days.

### 2.6. Adhesion Assay

Differentiated cells were infected with *S*. Typhimurium or EIEC (10^8^ CFU/mL) alone or in association with different *Lactococcus* strains (10^8^ CFU/mL). The assay was performed in three different ways: (i) competition, in which both bacterial strains (*Salmonella* or EIEC and *Lactococcus*) were added simultaneously to the cells and incubated for 2 h. In this way, it is possible to understand whether the bacteria compete for binding to the cells; (ii) inhibition, in which a 2 h pre-treatment with each *Lactococcus* was carried out, followed by the addition of *Salmonella* or EIEC (without removal of the *Lactococcus*) for a further 2 h. In this way, the ability of *Lactococcus* strains to preventatively protect the intestinal epithelium can be deduced; (iii) displacement, in which the cells were first infected with *Salmonella* or EIEC for 2 h, and then the pathogen was removed by removing the cell supernatant and fresh medium with *Lactococcus* added for an additional 2 h. This method aims at evaluating the ability of *Lactococcus* strains to remove pathogens from infected cells.

At the end of the experiment, the cell supernatants were removed and the infected cells were washed with PBS and lysed with 0.1% Triton X-100 solution. Lysates were serially diluted, plated on Hektoen enteric agar (HE), and incubated at 37 °C overnight to quantify the total number of viable cell-associated bacteria (CFU/mL). On this selective and differential medium, lactococci do not grow, whereas *Salmonella* and EIEC colonies are pigmented in black and red, respectively.

The adhesion efficiency was calculated as the ratio between the number of bacteria attached to the cells and the number of bacteria used for infection.

### 2.7. Production of Antimicrobial Substances

A spot-on-lawn assay was used to detect antimicrobial activity against the indicator bacteria reported above. Five microliters of LAB overnight cultures was spotted on ESTY agar plates and incubated for 24 h to allow colony growth. One hundred microliters of the indicator bacteria was added to six millilitres of soft appropriate media, poured on the above-described LAB spotted plates, and incubated at 37 °C. After 24 h of incubation, plates were checked for inhibition zones [41]. The agar well diffusion method was used to assess the production of antimicrobial substances from LAB strains. Overnight cultures of LAB were centrifuged, and the supernatants were collected and filtered through a 0.22 µm pore-size sterile filter (Millipore, Bedford, MA, USA). An aliquot of each supernatant was neutralized to pH 7.0 with 1 N NaOH. One hundred microliters of each bacterial suspension (10^6^–10^7^ cells/mL) were mixed with five millilitres of soft agar and poured over the plates. Wells of 6 mm in diameter were made into the inoculated plates and then filled with 100 µL of each supernatant. The plates were then incubated at 37 °C for 24 h. The antimicrobial activity of each LAB strain was evaluated by the development of inhibition halos around the wells [42]. All experiments were performed in triplicate and in three independent assays.

### 2.8. Safety Parameters

#### 2.8.1. Antibiotic Susceptibility Test

The *L. lactis* strains under study were tested for susceptibility towards vancomycin (30 µg), ampicillin (10 µg), gentamycin (10 µg), penicillin G (10 µg), and tetracycline (30 µg) by the Kirby–Bauer disc diffusion method [43]. After 24 h of incubation, inhibition zones were measured for each strain. Antimicrobial discs were supplied by Condalab (Madrid, Spain).

#### 2.8.2. Haemolytic Activity

Haemolytic activity was determined according to the protocol described by Maragkoudakis [44]. Colonies were streaked on blood agar plates and incubated for 48 h. Colonies were also examined seven days after incubation. *S. aureus* ATCC 6538 was used as a positive control for β-haemolysis, whereas *L. plantarum* WCFS1 was used as a positive control for γ-haemolysis. After incubation, colonies were checked for the presence of α/α (a small zone of greenish–brownish discoloration of the medium, indicating the reduction of haemoglobin to methaemoglobin), β/β (clear, colourless, or light-yellow zone surrounding the colonies depicting the total lysis of red blood cells), or γ/γ (with no change observed in the medium).

### 2.9. Statistical Analysis

Significant differences among experimental groups were assessed through a two-way ANOVA by using GraphPad Prism 8.0 for Windows (GraphPad Software, Boston, MA, USA, https://www.graphpad.com, accessed on 18 January 2024), and the comparison between the means by using Student’s *t*-test. Data are expressed as the mean ± standard deviation (SD) of three independent analyses. Statistical significance was defined as a two-tailed *p*-value of less than 0.05.

## 3. Results and Discussion

*Lactococcus lactis* subsp. *lactis* is one of the most important starter bacteria used for dairy production. However, to the best of our knowledge, only a few studies have analysed the probiotic characteristics of strains isolated from raw milk and dairy products [28,45]. Therefore, this study aimed to analyse the probiotic properties of nine strains of *L. lactis* subsp. *lactis* isolated from natural whey starter cultures of cow and buffalo milk for the production of artisanal cheeses [36].

### 3.1. Survivability in Simulated Gastric Juice and Bile Salts

Resistance to gastric pH and high bile concentrations are key features of probiotic strains that can withstand the unfavourable conditions of the gastrointestinal tract (GIT). The effects of simulated gastric juice and bile salts on the survival rate of the isolates after 3 h of incubation are reported in Table 1. All the strains maintained a high survival rate when exposed to gastrointestinal stress. Particularly, more than 90% of viable cells were detected in the presence of 0.3% pepsin at pH 3.0, with peaks of 98–100% for the A3, D1, D3, and I7 isolates (Table 1). Contrariwise, all the tested strains failed to survive in the presence of 0.3% (*w*/*v*) pepsin at pH 2.0 (Table 1). According to the latter result, to our knowledge, no data are reported on the survival of *Lactococcus* spp. at pH 2.0. The ability to survive at extremely acidic pH has been described for some LAB strains, mainly those belonging to the *Lactobacillus* and *Bifidobacterium* genera [46,47]. Furthermore, Tsigkrimani and co-workers (2022) reported that the survival rate of 189 LAB belonging to 10 different species drastically decreased after exposure to pH 2.0 (25.9%) compared with pH 3.0 (88.9%) [48]. Similarly, a greater than 2 log CFU/mL reduction in the survival of 116 LAB strains isolated from Serpa cheese at pH 2.0 was observed by Ruiz-Moyano et al. [49]. The ability of potential LAB probiotic strains to tolerate an acidic environment is important not only for overcoming GIT stresses, but also for their application as food supplements and for improving their survival in fermentation processes [50].

The physiological concentration of bile salts in the intestinal tract varies between 0.3 and 0.5% (*w*/*v*). Probiotics are expected to pass through the gut and survive in the presence of bile salts while exerting their beneficial effects. In this study, strain-specific differences were observed when the isolates were exposed to a simulated small-intestine environment (0.3% *w*/*v* bile salts at pH 8.0) (Table 1). In particular, the *L. lactis* subsp. *lactis* A5 and D3 strains showed the greatest sensitivity compared to the other strains analysed, with survival rates of 52% and 67%, respectively. In contrast, high resistance was found for the strains I1, I7, and A3 which showed 100, 89, and 85% resistance, respectively, to bile juices. The ability of LAB from different sources to tolerate bile in a strain-specific manner has been previously demonstrated [45,48].

### 3.2. Hydrophobicity and Aggregative Potential

Cell surface hydrophobicity was determined by the MATH method [40], which is based on the affinity of microorganisms to organic solvents. In this work, extraction with xylene was used. The hydrophobic value of the *Lactococcus* strains under study varied from 25.5 to 78.8% (Table 1). In particular, all strains tested showed hydrophobicity values higher than 50%, except for the strains B1, D1, and I1, which showed 33.9, 25.5, and 35.9%, respectively. Indeed, the hydrophobic potential is organism- and strain-specific and can be affected by the cell growth phase and surface chemistry of strains, as well as by the composition of the culture medium. Good hydrophobicity has already been shown for strains of different species, including *Lactobacillus* (43–79%), *Pediococcus* (51.3–79%), *Bifidobacterium* (39–87%), and *Lactococcus* (52–89.7%) [28,51,52]. It has often been reported that high hydrophobicity is a positive feature in terms of probiotic properties; however, there is no standard hydrophobicity value sought in bacteria [28]. Although the adhesion capacity is not always correlated with hydrophobicity, the evaluation of surface hydrophobicity is considered by many authors to be a necessary pre-test to define the adhesion capacity of probiotic bacteria to epithelial cells [28,53].

**Figure 1:**
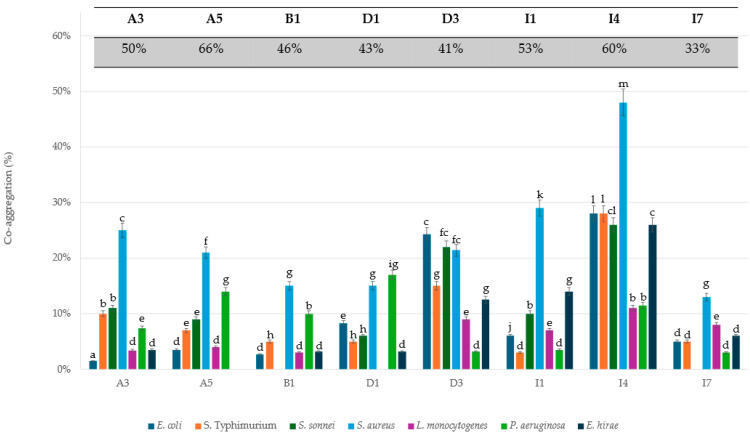
Co-aggregative properties between each probiotic strain and pathogens. Data are representative of three different experiments ± standard deviation (SD). In the grey box, the auto-aggregation percentage of the *L. lactis* strains, as shown in Table 1. ^a–m^ Different lowercase letters on the bar graph indicate significant differences (*p* < 0.001) in co-aggregation.

The microbial auto-aggregative ability is the capability of bacteria to form cellular aggregates that can positively affect their adhesion to the intestinal mucosa. In this study, the auto-aggregation capabilities of *L. lactis* strains and enteric pathogens were assayed. The *L. lactis* strains showed higher auto-aggregation values (ranging between 33.8% and 66.0%) (Table 1) than the foodborne pathogens, with values between 23.3% and 51.0%. The highest percentage of auto-aggregation was observed in the strain A5 (66.0%) among the LAB and *S. aureus* (51.0%) among the pathogens. To study the co-aggregation ability with pathogens, each LAB strain was tested with all the pathogens used in this study. According with the results of Taheri and co-workers (2009) [54], our data showed poor or no co-aggregation between LAB strains and pathogens (Figure 1). Even the strains that showed a good percentage of self-aggregation in pure cultures (Table 1) showed a drastic decrease in aggregation capacity when mixed in co-cultures. For instance, 21.0% co-aggregation was observed between A5 and *S. aureus*, which are both well-performing strains for auto-aggregation. Among the *Lactococcus* strains analysed, I4 showed the highest co-aggregation value (48%) with the foodborne pathogen *S. aureus*. Moreover, the I4 strain also showed a 28% co-aggregation value with the enteric pathogens *E. coli* and *S. typhi*, and 26% values with *S. sonnei* and *E. hirae*.

Taken together, these data show that all tested strains have cell surface hydrophobicity and a high capacity for auto-aggregation, characteristics that are typically associated with intestinal bacterial adhesiveness [52,55]. More specifically, the strains A3, A5, I4, and I7 had higher values of both auto-aggregation and hydrophobicity, suggesting that these strains have gut epithelial colonization ability. Among these, I4, as mentioned above, is the only one that shows a high co-aggregation value with the tested pathogens. However, the significance of co-aggregation in the scientific community remains an open issue. According to some researchers, lower levels of co-aggregation with pathogens are required for probiotics to prevent biofilm formation and to further minimize pathogen colonization of the intestinal tract [55]. On the contrary, other researchers have stated that the co-aggregation of probiotic bacteria with pathogens is a positive feature, because it is one of the possible ways to eliminate pathogens from the intestinal tract [56].

Given the poor or lack of co-aggregation ability of the strains under investigation, further studies were performed to investigate their potential to inhibit pathogens. Indeed, there are different mechanisms by which probiotic bacteria can inhibit pathogens’ adhesion [56]. These may include the ability of probiotics to stimulate the secretion of mucin glycoproteins and antimicrobial proteins (defensins) by intestinal epithelial cells (IECs), which help to eliminate commensals or pathogens from the mucus layer or the production of antimicrobial substances such as bacteriocins. Furthermore, most probiotics can compete with enteric pathogens for adhesion sites in the mucus layer or IECs, thereby preventing harmful colonization and enhancing barrier function [57]. In this context and on the basis of the previous analysis, the four best-performing *L. lactis* strains (A3, A5, I4, and I7) were chosen to test their ability to interfere with the adhesion of intestinal pathogens to epithelial cells, as described in the next paragraph.

### 3.3. Activity of L. lactis Strains against S. Typhimurium and EIEC Adhesion to Epithelial Cells

The adhesion of probiotics to the intestinal epithelium is essential for their beneficial effects. They exert a positive regulation of the immune system, interacting with the cells of the immune system present in the gut-associated lymphoid tissue and counteracting the onset of massive inflammatory phenomena [58]; furthermore, they are able to produce bioactive products, such as antimicrobial peptides [55], short-chain fatty acids [59], and other metabolites, in proximity to the host cells, and to strengthen the gastrointestinal barrier through the production of tight junctions. In particular, by adhering to the intestinal epithelium, probiotics protect against pathogen attack through a competitive exclusion mechanism [60]. However, probiotics must survive the technological processes necessary for their commercialization. Exposure to high temperatures or drying processes could reduce the ability of probiotics to adhere to intestinal cells [61].

**Figure 2:**
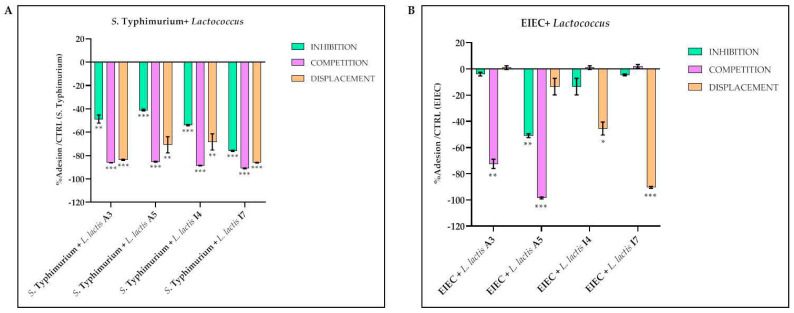
Inhibition, competition, and displacement assays: percentage of reduction in *S.* Typhimurium (**A**) and EIEC (**B**) adhesion to epithelial cells in the presence of *Lactococcus* strains. Data are representative of three different experiments ± SD. Significant differences are indicated by * *p* < 0.05, ** *p* < 0.01, *** *p* < 0.001.

In our experimental system, the results obtained for the *S*. Typhimurium adhesion assay showed that all *L. lactis* strains were able to significantly reduce the adhesion of *S*. Typhimurium to intestinal epithelial cells, particularly in the competition assay. Among the four strains tested, the most effective was I7, showing adhesion reductions of 76%, 91%, and 86% in inhibition, competition, and displacement, respectively (Figure 2A, Table 2).

In the EIEC adhesion assay, different strains of *L. lactis* isolates acted in different ways. As shown in Figure 2B and Table 2, A3 was able to significantly reduce bacterial adhesion only in the competition assay (72.5%); A5 acts in the inhibition assay but particularly in the competition assay (51%, and 98.5%, respectively); I4 and I7, particularly the latter, work only in the displacement assay (45.5% and 90.5%, respectively). Overall, the best-performing strain concerning this parameter is A5, although the low percentage of resistance to bile (52%) does not include it among the best-performing strains.

Taken together, these data are in full agreement with previous studies on the interactions between probiotics and gastrointestinal pathogens [60,62,63,64,65], where it is clearly reported that the effectiveness of probiotics in inhibiting, competing, or displacing the pathogens is strictly variable and related to the species of both the probiotic and the pathogen. It has also been widely demonstrated that the mechanisms underlying the inhibition and competition processes are different from those involved in that of displacement: in fact, the inhibition could be due either to the co-aggregation between the probiotic and the pathogen or to a mechanism of competitive exclusion for the binding sites which, being occupied by the probiotic during pre-treatment, are no longer available for the pathogen; during competition, probiotics and pathogens can compete directly both for the binding sites and for nutrient availability in the environment; in the case of displacement, however, the ability to “detach” pathogens from their binding sites could be due both to the production of bacteriocins and/or to a stimulation of the immune system.

### 3.4. Detection of Antimicrobial Activity

The inhibitory effects of LAB may be due to natural protective organic acids, hydrogen peroxide, diacetyl, bacteriocins, and specific substances [66]. The LAB antimicrobial molecules production represents an important feature in both clinical and food fields, as it can inhibit pathogens and food-degrading microorganisms that shorten the shelf life of food products [67,68,69].

In this work, the spot on lawn method was used as a first approach to detect the antimicrobial activity of the LAB under study (see Section 2). After 24 h of incubation, no inhibition zones were detected, indicating that none of the tested strains were able to inhibit the growth of the tested pathogens. Due to the negative results obtained from this assay, the agar well diffusion method was also performed. In this experiment, all the *L. lactis* strains, except I1, were able to inhibit the growth of the *S. sonnei* strain, but the bactericidal effect was completely lost when the supernatants were neutralized, indicating that the antimicrobial activity could be due to the fermentative production of organic acids rather than the presence of bacteriocins. However, the presence of an antimicrobial activity is rare and often difficult to detect. Mìnguez and co-workers (2012) reported that only 52 out of all 169 LAB isolates from infant faeces showed an inhibition zone higher than 10 mm against *Escherichia coli* and *Listeria innocua* [70]. Similar results have been obtained with a variety of LAB isolates found ineffective against several species of pathogens [71,72]. It has been reported that the diffusion, aggregation, and concentration of bacteriocins, and cellular proteolytic activity are important factors that can interfere with the measurement of bacteriocin activity in agar well diffusion tests [73,74]. Indeed, the detection of LAB antimicrobial activity could depend on the indicator strains and methods used in the experiments. Because some bacteriocins have very narrow targets, it is important to select sensitive indicator strains. It is also possible that several bacteriocins encoded by chromosomes or plasmids are not expressed under certain conditions. It has been reported that the production of bacteriocins depends on the composition of the medium and the culture conditions [75]. The optimization of this process often requires the use of bioreactors, which allow for fine control of the different parameters that influence the growth and production of bacteriocins. Further studies are therefore needed to investigate the antimicrobial activity of these newly isolated strains [76].

### 3.5. Antibiotic Susceptibility Test

The isolated strains were tested for antibiotic susceptibility, an important parameter for a microorganism to be considered safe for human and animal administration. An antibiotic susceptibility test was performed according to the agar diffusion method (Kirby–Bauer test) against five antimicrobial agents: ampicillin (AM), penicillin (P), gentamicin (CN), vancomycin (VA), and tetracycline (TE). The results of these assays are reported in Table 3 and are expressed as resistant (R), sensible (S), and intermediate (I). All isolates were found to be very susceptible to ampicillin, penicillin, tetracycline, and vancomycin with inhibition zones ranging from 25 to 36 mm. Only three of the eight tested strains showed resistance to gentamicin. In general, *L. lactis* strains are susceptible to broad-spectrum antibiotics and β-lactam antibiotics, which are effective against Gram-positive bacteria. Instead, resistance to gentamicin and other aminoglycosides in dairy *L. lactis* strains has often been reported [28]. Poelarends and co-worker [77] demonstrated that the presence of the LmrA transporter in *L. lactis* is associated with the innate resistance to clinically relevant antibiotics, including aminoglycosides [28].

### 3.6. Haemolytic Activity

According to the FAO/WHO (2002) [78] guidelines for probiotic evaluation, safety is a crucial aspect to consider, and the absence of haemolytic activity is one of the criteria for selecting probiotic strains. This is because haemolytic activity indicates the potential virulence of the bacteria. Strains lacking haemolytic activity are considered safe and non-virulent, and the lack of haemolysin ensures that opportunistic virulence does not appear among strains [71]. None of the tested strains showed haemolytic activity on blood agar indicating their non-virulent nature *in vitro*.

## 4. Conclusions

This study aimed to assess the probiotic potential of eight *L. lactis* strains, previously isolated from natural way starter cultures. All the strains exhibited a high survival rate under gastrointestinal stress, with values ranging from 95% to 100% for gastric stress and 52% to 100% for bile salts stress. An analysis of hydrophobicity (25.5–78.8%) and auto-aggregation (33–66.0%) revealed that all strains had a good adhesive potential but showed poor or no ability to aggregate with pathogens. Although no strain produced molecules with antimicrobial activity, under the conditions used, most strains showed the ability to interfere with the binding of pathogens to the intestinal epithelium. In addition, the newly isolated *L. lactis* strains were tested for safety parameters, such as antibiotic susceptibility and haemolytic activity, and all strains exhibited safe characteristics. As expected, the properties assessed in this study were found to be strain-dependent. The results showed that the strain I7 was the most promising, as it demonstrated the ability to survive in GI environments (99.0% and 89% acid and bile tolerance, respectively), exhibited 64% and 33% hydrophobicity and auto-aggregation, respectively, and interfered with the binding of *S.* Typhimurium (adhesion reduction ranging from 76 to 91%, depending from the assay) and EIEC to the intestinal epithelium (displacement 90.5%). The potential use of probiotics in the treatment of *E. coli* gut infections makes the latter data particularly interesting. Although further studies are needed to confirm the safety and probiotic properties of these strains, these results suggest that the new isolated strains of *L. lactis* could be considered for use as probiotics.

## Figures and Tables

**Table 1:** Survival of *L. lactis* strains under simulated GIT conditions and cell surface properties. Acid tolerance (0.3% pepsin, pH 3.0) and bile tolerance (0.3% bile salts, pH 8.0) were assessed after 3 h of incubation.

		Survival to GI Conditions * (% logCFU/mL)		Cell Surface Properties * (%)	
STRAINS	Acid Tolerance (pH 2.00)	Acid Tolerance (pH 3.00)	Bile Tolerance (pH 8.00)	Hydrophobicity	Auto-Aggregation
A3	ND	100 ± 0.01 ^a^	85.0 ± 0.04 ^bd^	68.7 ± 0.01 ^A^	50.0 ± 0.4 ^G^
A5	ND	94.0 ± 0.06 ^a^	52.0 ± 0.02 ^c^	78.8 ± 0.03 ^B^	66.0 ± 0.4 ^H^
B1	ND	98.0 ± 0.03 ^a^	81.0 ± 0.02 ^db^	33.9 ± 0.01 ^C^	46.4 ± 0.1 ^I^
D1	ND	98.0 ± 0.02 ^a^	71.0 ± 0.02 ^ed^	25.5 ± 0.02 ^D^	43.3 ± 0.2 ^J^
D3	ND	98.0 ± 0.02 ^a^	67.0 ± 0.01 ^f^	66.5 ± 0.02 ^EA^	41.0 ± 0.1 ^K^
I1	ND	100 ± 0.01 ^a^	100 ± 0.01 ^g^	35.9 ± 0.01 ^C^	53.0 ± 0.5 ^G^
I4	ND	95.0 ± 0.01 ^a^	77.0 ± 0.01 ^de^	51.6 ± 0.01 ^F^	60.0 ± 0.3 ^L^
I7	ND	99.0 ± 0.02 ^a^	89.0 ± 0.01 ^bd^	64.0 ± 0.02 ^E^	33.0 ± 0.1 ^M^

* Results of three independent experiments ± SD. ^a–g^ and ^A–M^ Data in columns with different superscripts differ (*p* < 0.001).

**Table 2:** Average values of colony-forming units/mL (CFU/mL) of *S*. Typhimurium and EIEC after adhesion assay with *Lactococcus* strains. The average CTRL values are 4.1 × 10^8^ for *S*. Typhimurium and 5.5 × 10^6^ for EIEC. Data are representative of three different experiments.

	Inhibition	Competition	Displacement
*S.* Typhimurium + A3	2.1 × 10^8^	5.7 × 10^7^	6.7 × 10^7^
*S.* Typhimurium + A5	2.4 × 10^8^	6.0 × 10^7^	1.2 × 10^8^
*S.* Typhimurium + I4	1.9 × 10^8^	4.7 × 10^7^	1.3 × 10^8^
*S.* Typhimurium + I7	1.0 × 10^8^	3.7 × 10^7^	5.7 × 10^7^
EIEC + A3	5.7 × 10^6^	1.5 × 10^6^	5.4 × 10^6^
EIEC + A5	2.7 × 10^6^	8.3 × 10^4^	4.7 × 10^6^
EIEC + I4	4.7 × 10^6^	5.4 × 10^6^	3.0 × 10^6^
EIEC + I7	5.4 × 10^6^	5.5 × 10^6^	5.0 × 10^5^

**Table 3:** Antibiotic sensitivity test (AM, ampicillin; P, penicillin; CN, gentamicin; VA, vancomycin; TE, tetracycline; S, sensitivity; R, resistance).

Strains	AM 10 µg	P 10 µg	CN 10 µg	TE 30 µg	VA 30 µg
A3	S	S	S	S	S
A5	S	S	R	S	S
B1	S	S	S	S	S
D1	S	S	S	S	S
D3	S	S	S	S	S
I1	S	S	R	S	S
I4	S	S	R	S	S
I7	S	S	S	S	S

## Data Availability

The original contributions presented in the study are included in the article, further inquiries can be directed to the corresponding author.
